# Surgical Treatment of Thoracic Outlet Syndrome Secondary to Clavicular Malunion

**DOI:** 10.4055/cios.2009.1.1.54

**Published:** 2009-02-06

**Authors:** Moon Jib Yoo, Joong Bae Seo, Jong Pil Kim, Ju Hong Lee

**Affiliations:** Department of Orthopaedic Surgery, Dankook University College of Medicine, Cheonan, Korea.

**Keywords:** Clavicle, Malunion, Thoracic outlet syndrome, Scalenetomy

## Abstract

According to the literature, thoracic outlet syndrome (TOS) secondary to the malunion of displaced fractures of the clavicle is rare. Various surgical methods, including simple neurolysis, resection of the first rib or clavicle and corrective osteotomy, have been reported. We report a case of TOS secondary to malunion of the clavicle that was treated by an anterior and middle scalenectomy without a rib resection.

According to the literature, thoracic outlet syndrome (TOS) secondary to a malunion of displaced fractures of the clavicle is rare.^1^-7) The etiology of TOS caused by malunion of the clavicle is an inferior protrusion of bone that directly compresses the underlying neurovascular structures in the costoclavicular space. A variety of surgical treatments have been reported, including resection of the callus, resection of all or a part of the clavicle, resection of the undersurface of the clavicle and corrective osteotomy (Table 1).

The following TOS case produced by malunion of the clavicle was treated completely with an anterior and middle scalenectomy, which are soft-tissue procedures, without a rib resection.

## CASE REPORT

A 40-year-old male laborer visited our outpatient clinic complaining of paresthesia and weakness in his left upper extremities. He had fallen off a truck 9 months earlier and sustained a fracture on the middle third of his left clavicle. He was treated by a local orthopedist using a figure-8 bandage, which was removed 6 weeks later. At the time of the injury and after removing the bandage, he did not report any neurological problems. He returned to his job 3 months after the injury. However, seven months after the injury, he began to experience pain and paresthesia along the medial aspect of his left arm and ulnar digits. In addition, he reported progressive weakness in his left extremities, and was unable to work.

The physical examination revealed a visible, palpable, and non-tender prominence over the middle third of the right clavicle. The patient demonstrated a full range of active and passive motion in the left shoulder but experienced pain and paresthesia, which was exacerbated during the Adson and 3-minute Roos tests.8)

**Fig. 1:**
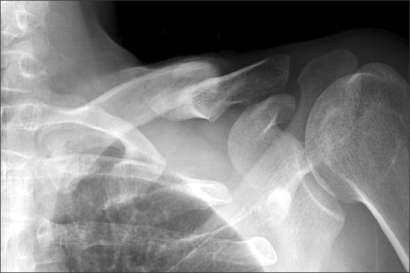
Anteroposterior radiograph of the left clavicle 9 months after the injury. A typical clavicular malunion deformity resulting from that the proximal fragment was displaced superiorly, while the distal fragment was displaced inferiorly, translated medially, and rotated anteriorly after the midshaft clavicular fracture, is demonstrated.

**Fig. 2:**
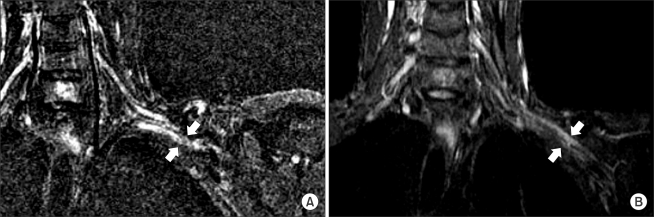
Coronal T2-weighted magnetic resonance images of the left brachial plexus. (A) The preoperative image shows that the left brachial plexus was compressed by the malunited clavicle in the costoclavicular space. (B) The postoperative image at the follow-up performed 16 months after surgery shows that the distorted brachial plexus lined up in a straight line in contrast to the alignment observed before surgery.

Plain radiographs showed a typical deformity of malunion of the clavicle with overlapping proximal and distal fragments (Fig. 1). Magnetic resonance imaging of the shoulder showed an abnormal signal on the coronal T2-weighted image of the left brachial plexus, which was compressed by the inferiorly protruded fragment of the clavicle (Fig. 2A). A nerve conduction study and electromyography demonstrated a decreased velocity and amplitude of ulnar nerve conduction from Erb's point to the axilla. In addition, increased insertional activities and positive sharp waves were observed in the C8 and T1 innervated muscles. A diagnosis of neurogenic TOS secondary to clavicular malunion was made based on the patient's history, physical examination, imaging studies and electrodiagnostic tests. We planned to perform an anterior and middle scalenectomy with a first rib resection.

**Fig. 3:**
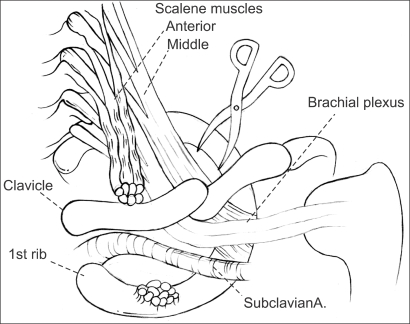
Schematic diagram of a supraclavicular scalenectomy. After exposing the anterior and middle scalene muscles and brachial plexus via the supraclavicular approach, the distal part of the anterior scalene muscle was divided completely followed by a middle scalenectomy.

With the patient under general anesthesia, an anterior and middle scalenectomy was performed via the supraclavicular approach (Fig. 3). We did not perform the planned first rib resection because the callus and malalignment of the clavicle did not narrow the costoclavicular space and did not compress the neurovascular structures directly. The brachial plexus beneath the clavicle was covered with a normal padding of fat that did not adhere to any of the structures surrounding it.

Immediately after surgery, the pain and paresthesia in the left upper extremities had improved considerably. Ten weeks after surgery, another nerve conduction study and electromyography revealed complete symptom relief. The patient was asymptomatic 16 months after surgery. In addition, magnetic resonance imaging, which was carried out 16 months after surgery, showed that the brachial plexus, distorted due to compression from a malunited clavicle, lined up in a straight line in contrast to the alignment observed before surgery (Fig. 2B).

## DISCUSSION

Mulder et al.4) resected the middle half of the clavicle and Enker and Murthy2) performed a total clavicle resection. A partial or total resection of the clavicle will cause some degree of instability. Rowe5) resected an excessive callus formation, and Fujita et al.3) resected an inferiorly protruded bone using pulse-wave measurements of the fingertips. Although less invasive than a partial or total resection, these procedures are technically difficult when there is callus formation that is intimately related to the neurovascular structures. Connolly and Ganjianpour1) resected the malformed bone and excess callus from a double osteotomized clavicle and reimplanted the reduced segment into the osteotomy site. A corrective clavicular osteotomy that is fixed internally can result in some complications, including nonunion, implant failure and excessive callus formation.

In our case, only an anterior and middle scalenectomy was performed by avoiding the procedures associated with the costoclavicular space, including resection of the callus, first rib, or clavicle. Nevertheless, according to a second nerve conduction study and electromyography, the patient obtained complete relief from his symptoms without complications. In addition, magnetic resonance imaging carried out 16 months after surgery, showed that the distorted brachial plexus due to compression by the malunited clavicle lined up in a straight line, which is in contrast to the preoperative alignment.

Kitsis et al.6) reported the postoperative results showing that 14 patients with delayed development of brachial compression symptoms after the clavicular fractures had been treated by a callus excision, simple neurolysis, internal fixations for nonunions, or corrective clavicular osteotomy. However, according to that study, the majority of the patients experienced some degree of residual symptoms. In our patient, the callus and malalignment of the clavicle did not compress the neurovascular structures, and the costoclavicular space was large enough to mobilize the brachial plexus freely after the scalenectomy. The brachial plexus beneath the clavicle was covered with a normal padding of fat that did not adhere to any of the structures surrounding it. This suggests that compression of the brachial plexus and subclavian vessel in the costoclavicular space can be corrected by a resection of the scalene muscles, which are the structural components of the costoclavicular boundary. It also suggests that the main pathology of neurogenic TOS was in the scalene muscles due to a tethering effect, which might be a cause of the residual symptoms if not corrected.

Because TOS due to malunion of the clavicle is caused by an inferiorly protruded bone that compresses directly the underlying neurovascular structures in the costoclavicular space, most surgical treatments have focused on the costoclavicular space. However, our case shows that compression of the brachial plexus caused by malunion of the clavicle can be treated successfully by anterior and middle scalenectomy without the procedures to correct the deformed costoclavicular space, including a first rib resection.

## Figures and Tables

**Table 1:**
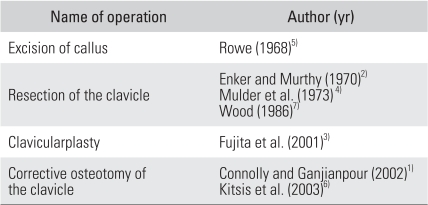
Surgical Treatments of Thoracic Outlet Syndrome Secondary to a Clavicular Malunion: Literature Review
